# Editorial: Unlocking the potential of prenatal MRI: advances in fetal brain, heart, and placenta imaging

**DOI:** 10.3389/fcvm.2026.1836505

**Published:** 2026-04-13

**Authors:** Mehdi H. Moghari, Christopher K. Macgowan, Christopher W. Roy, Maria Chase, Nita R. Chaudhuri, Jai P. Udassi

**Affiliations:** 1 Department of Pediatrics, West Virginia University, Morgantown, WV, United States; 2 Department of Cardiology, West Virginia University Medicine Children’s Hospital, Morgantown, WV, United States; 3 Translational Medicine Program, The Hospital for Sick Children, University of Toronto, Toronto, ON, Canada; 4 Department of Medical Biophysics, Faculty of Medicine, University of Toronto, Toronto, ON, Canada; 5 Lausanne University Hospital (CHUV) and University of Lausanne (UNIL), Lausanne, Switzerland

**Keywords:** fetal brain MRI, fetal heart MRI, fetal magnetic resonance imaging (MRI), fetal placenta MRI, magnetic resonance imaging (MRI)

## 1 Introduction

For decades, fetal echocardiography has stood as the cornerstone of prenatal cardiac diagnosis. Its portability, real-time capability, and superior temporal resolution have made it indispensable for prenatal screening and longitudinal surveillance. Yet even in expert hands, echocardiography remains constrained by maternal body habitus, fetal position, oligohydramnios, advanced gestational age all leading to limited acoustic windows. These limitations have sustained interest in fetal cardiovascular magnetic resonance imaging (MRI) as a complementary modality. Historically, however, technical barriers including the absence of reliable cardiac gating, fetal bulk motion, maternal respiration, and low signal-to-noise ratio have confined fetal cardiovascular MRI largely to the research domain (1, 2).

Importantly, fetal MRI offers physiologic and hemodynamic insights that are fundamentally inaccessible after birth. Prenatal quantification of brain and cardiac volumes, vessel flow, and fetoplacental circulation can meaningfully influence delivery planning, immediate neonatal management, and surgical strategy, particularly in complex congenital heart disease (CHD). The value of fetal MRI therefore lies not in imaging earlier, but in informing perinatal decision-making and potentially improving postnatal outcomes.

Moreover, addressing the technical challenges inherent to fetal MRI carries broader translational implications. The need for rapid, motion-robust, non-contrast imaging directly informs innovation in pediatric and adult MRI, where contrast avoidance, free-breathing acquisitions, and accelerated imaging remain pressing priorities.

The seven manuscripts in this Research Topic collectively signal a paradigm shift. Rather than addressing isolated bottlenecks, they represent a unified vision: transforming fetal MRI into a clinically deployable, multiparametric imaging platform that spans cardiac, placental, and neurodevelopmental domains, particularly in fetuses with CHD, in whom the development of the heart, placenta, and brain could be codependent. Topics covered include improvements to how fetal MRI data are acquired, using novel hardware platforms and acquisition strategies to provide more robust measures of cardiovascular function and tissue structure. These are complemented by studies demonstrating advanced postprocessing approaches, leveraging deep learning to reduce image noise and improve resolution in fetal MRI applications. Finally, work is presented using MRI to improve our understanding of normal fetal development, and the consequences disease. Collectively, this body of work highlights a rapidly maturing field and points toward a promising future in which fetal MRI plays an increasingly central role in managing at-risk pregnancies.

## 2 Complementing echocardiography: structured cardiac assessment with DUS-gated MRI

Hergert et al. prospectively evaluated the diagnostic performance of Doppler Ultrasound (DUS)-gated (3) fetal cardiovascular MRI at 3 T using the standardized five axial cardiac views on 29 pregnant women. Benchmarking against fetal echocardiography, the authors demonstrated that MRI can reproducibly acquire abdominal situs, four-chamber, left ventricular outflow tract, right ventricular outflow tract, and three-vessel views with superior image quality.

MRI achieved 88% sensitivity and 100% specificity for detecting cardiac abnormalities, compared with 100% for echocardiography. Quantitative structural measurements showed no significant inter-modality differences. Notably, MRI performed particularly well in evaluating the right ventricular outflow tract, an area with limited visualization at times on ultrasound. In contrast, valvar abnormalities remained better characterized by echocardiography.

Clinically, DUS-gated MRI is best positioned as an adjunct, particularly when ultrasound windows are compromised. Despite limitations including modest sample size and motion susceptibility, this work provides compelling prospective evidence that gated fetal MRI can support structured CHD evaluation and enhance prenatal diagnostic confidence.

## 3 Re-thinking field strength: feasibility of low-field (0.55 T) fetal cardiac MRI

Zhang et al. challenged the long-held assumption that high magnetic field strength is essential for fetal cardiovascular MRI. Their study demonstrated the feasibility of comprehensive structural and flow imaging on 23 pregnant women at 0.55 T through careful optimization of balanced steady-state free precession cine and phase-contrast gradient-echo sequences.

While low-field imaging inherently reduces signal-to-noise ratio, it offers compensatory advantages highly relevant to fetal imaging: reduced susceptibility artifact, improved B_0_ homogeneity, lower specific absorption rate, decreased acoustic noise, and more favorable T_2_/T_1_ contrast behavior (4–6). In addition, the wider bore of whole-body 0.55 T scanners enhance maternal comfort.

The authors further demonstrated reliable quantification of major fetal vessel flow using metric-optimized gating (7), with good agreement relative to DUS, particularly at lower flow conditions. Low-field systems may be specifically advantageous in late gestation, when ultrasound windows degrade and patient comfort becomes critical. Although longer scan times and lower intrinsic signal-to-noise ratio remain limitations, this work establishes low-field MRI as a viable platform that may broaden global access to fetal cardiovascular imaging while reframing field strength as a modifiable and not limiting parameter.

## 4 Deep learning reconstruction: enhancing image fidelity

Vollbrecht et al. addressed the persistent barrier of limited image quality in fetal cardiovascular MRI by evaluating deep learning (DL)–based denoising reconstruction in DUS-gated cine imaging. In 23 fetuses with CHD, convolutional neural network reconstruction was compared with conventional compressed sensing.

DL reconstruction nearly doubled apparent signal-to-noise ratio and contrast-to-noise ratio, significantly improving blood–myocardium contrast and endocardial border definition. These gains translated into higher reader confidence across key structures, including ventricles, valves, great vessels, pulmonary veins, and the aortic arch.

Artifact burden remained comparable between techniques; however, the study highlights a critical note: DL denoising does not correct motion. In some cases, enhanced contrast made motion artifacts more conspicuous, reinforcing the need for cautious interpretation of AI-enhanced images. A notable strength is clinical deployability. Reconstruction was performed under one minute and did not rely on fetal-specific training datasets, suggesting vendor-integrated DL pipelines can be implemented without workflow disruption.

By shifting focus from gating feasibility to reconstruction, this work establishes DL denoising as a complementary strategy in fetal cardiovascular MRI, improving interpretability without extending acquisition time.

## 5 Placental MRI: the missing physiologic link

Sadiku et al. expanded the cardiovascular paradigm of this Research Topic beyond the fetal heart, positioning the placenta as a central determinant of fetal hemodynamics and growth. Their review synthesized advanced multiparametric placental MRI techniques spanning perfusion, oxygenation, microstructure, and metabolism.

Methods such as arterial spin labeling, diffusion imaging, BOLD, quantitative susceptibility mapping, and MR spectroscopy enable physiologic interrogation far beyond morphologic assessment. In fetal growth restriction, these tools reveal reduced placental volume, impaired perfusion, and altered oxygenation as potential biomarkers for earlier detection of dysfunction.

The review is compelling in CHD, where mounting evidence suggests shared developmental pathways between placental and cardiac abnormalities. Distinct perfusion and oxygenation patterns across CHD subtypes support the concept of a disrupted fetoplacental unit rather than an isolated cardiac defect. Integrating placental metrics with fetal cardiovascular imaging may ultimately enable holistic prenatal risk stratification and refined perinatal management.

## 6 Super-resolution MRI for corpus callosum assessment

Lamon et al. evaluated 3D super-resolution (SR) fetal brain MRI for corpus callosum assessment on 57 fetuses between 21 and 35 weeks gestation. Compared with conventional T_2_-weighted MRI and ultrasound, SR reconstruction demonstrated superior reliability and reduced missing data across callosal sub-segments.

SR closely matched ultrasound measurements while significantly improving visualization of posterior structures (the body and splenium), where conventional MRI frequently fails. corpus callosum length, rostrum, and genu remained more challenging, reflecting inherent anatomic and resolution constraints. Importantly, the authors provide novel normative biometric charts for segmental callosal heights and area, filling a major gap in MRI-based fetal neuroanatomy. SR enabled evaluation in most partial agenesis cases, highlighting utility in borderline or complex presentations.

Clinically, SR MRI serves as a powerful adjunct when ultrasound is limited or inconclusive. When combined with diffusion imaging or tractography, it may support future classification systems linking commissural structure with neurodevelopmental prognosis specifically for fetuses with CHD.

## 7 Sex-specific neurodevelopment on in-utero diffusion MRI

Ren et al. leveraged diffusion-weighted imaging in 200 fetuses to characterize sex-specific brain microstructural development. By quantifying apparent diffusion coefficient trajectories across multiple regions, they identified subtle but meaningful developmental differences.

Male fetuses demonstrated higher ADC values in occipital and temporal white matter and cerebellar hemispheres in the third trimester, with cerebellar differences remaining significant after correction. Accelerated ADC decline in males suggests faster maturation trajectories, aligning with prior morphometric observations.

These findings reinforce the importance of sex-specific normative data when interpreting fetal MRI. By providing quantitative microstructural benchmarks, the study advances precision prenatal neuroimaging and informs individualized developmental assessment.

## 8 Rapid magnetization transfer imaging of fetal myelination

Sadanand et al. introduced a rapid magnetization transfer (MT) MRI technique, termed yarnball MT, for in-utero assessment of fetal brain microstructure and early myelination. The proposed sequence achieved whole-uterus coverage within a single 13–28-second breath hold. Phantom validation and pilot *in-vivo* studies demonstrated sensitivity to semisolid macromolecular content. MT contrast revealed expected maturation patterns in the posterior limb of the internal capsule, corpus callosum, basal ganglia, and pons, features often occult on standard imaging.

Complementing diffusion MRI, MT imaging probes myelin and macromolecular composition, offering a distinct window into fetal neurodevelopment. Together, these modalities signal a shift toward multimodal microstructural fetal brain imaging.

## 9 Conclusion

Collectively, the manuscripts in this Research Topic chart a coherent trajectory for fetal MRI, from technical feasibility to clinical translation. Advances in cardiac gating, field strength optimization, DL–based reconstruction, placental physiology, and neurodevelopmental imaging converge toward a single vision: comprehensive, non-contrast, motion-robust prenatal MRI capable of informing real-time perinatal care, particularly for fetuses with CHD, in whom the heart, placenta, and brain could influence one another.

As these innovations continue to mature, fetal MRI is poised to evolve from an adjunctive research tool into an integrated pillar of precision prenatal medicine, reshaping how we diagnose disease, stratify risk, and ultimately care for fetuses with cardiovascular, placental, and neurodevelopmental disorders.
